# Indirect comparison of novel Oral anticoagulants among Asians with non-Valvular atrial fibrillation in the real world setting: a network meta-analysis

**DOI:** 10.1186/s12872-019-1165-5

**Published:** 2019-07-31

**Authors:** Jianchao Zhang, Junnan Tang, Xiaolin Cui, Bo Wang, Mengsen Bu, Yan Bai, Kai Wang, Jiacheng Guo, Deliang Shen, Jinying Zhang

**Affiliations:** 1 Department of Cardiology, The First Affiliated Hospital of Zhengzhou University, NO.1 Eastern Jianshe Road, Zhengzhou, 450052 Henan China; 2 Henan Province Key Laboratory of Cardiac Injury and Repair, Zhengzhou, 450052 Henan China; 3 Department of Orthopaedic Surgery, University of Otago, Christchurch, 8011 New Zealand

**Keywords:** Novel Oral anticoagulants, Non-valvular atrial fibrillation, Asian patients, Network meta-analysis

## Abstract

**Background:**

The development of novel oral anticoagulants (NOACs) has changed the landscape of non-valvular atrial fibrillation (NVAF) management. In this study, the effectiveness and the safety of several NOACs were evaluated in a real-world setting among Asian patients with NVAF.

**Methods:**

The literature search was conducted crossing different databases including Embase, MEDLINE, and the Cochrane Library from inception through March 1, 2019, for studies which included real-world perspectives comparing the individual NOACs with each other or with warfarin among Asians with NVAF. The primary outcomes were defined as stroke or systemic embolism (SSE) and major bleeding; ischemic stroke, all-cause death as well as intracranial bleeding were classified as the secondary outcomes.

**Results:**

From sixteen real-world studies, a total of 312,827 Asian patients were included in this analysis. In comparison with warfarin, the utilization of apixaban, dabigatran, and rivaroxaban significantly lowered the risk of major bleeding (apixaban: HR 0.47, 95%CI 0.35–0.63; dabigatran: HR 0.59, 95%CI 0.47–0.73; rivaroxaban: HR 0.66, 95%CI 0.52–0.83) and lessened the all-cause death rate (apixaban: HR 0.29, 95%CI 0.16–0.52; dabigatran: HR 0.40, 95%CI 0.27–0.60; rivaroxaban: HR 0.42, 95%CI 0.28–0.65). Apixaban (HR 0.59; 95%CI 0.40–0.85) reduced the possibility of ischemic stroke when compared against dabigatran. Rivaroxaban showed a higher chance of causing an ischemic stroke (HR 1.61; 95%CI 1.08–2.41) and major bleeding (HR 1.39; 95%CI 1.02–1.90) than Apixaban.

**Conclusions:**

Apixaban, dabigatran and rivaroxaban were more effective than warfarin on reducing the risks of stroke and haemorrhage; meanwhile, apixaban was likely to lower the risk of major bleeding comparing to rivaroxaban.

**Trial registration:**

PROSPERO registry number: CRD42018086914.

**Electronic supplementary material:**

The online version of this article (10.1186/s12872-019-1165-5) contains supplementary material, which is available to authorized users.

## Background

Non-valvular atrial fibrillation (NVAF) is the most common arrhythmia associated with severe thromboembolic events. Stroke caused by NVAF often can cause higher immobility and mortality than other stroke risk factors [1]. Furthermore, the prevalence of atrial fibrillation (AF) is on the rise across the world especially in Asia [2], where there is a large and rapidly ageing population. Therefore, stroke prevention is crucially important for Asian patients with NVAF [3]. Although it has been used to prevent stroke for years, warfarin is still underused and under-dosed in Asian patients, and the quality of international normalized ratio (INR) control is substandard in Asia compared with its western counterpart [4, 5]. This may be due to the fact that Asian patients are more sensitive to warfarin with a narrow INR range, accompanied by a higher risk of hemorrhagic complications [6]. As a result, low-intensity warfarin is often prescribed in clinical practice which may contribute to the increasing risk of embolism.

Different from warfarin, novel oral anticoagulants (NOACs) are lower risk and are excellent in preventing stroke and limiting haemorrhage especially intracranial haemorrhage [7, 8]. Moreover, a recent meta-analysis has suggested that NOACs are more effective and safer among Asian patients than non-Asians in terms of complications such as stroke or systemic embolism (SSE) and major bleeding [9], which indicates that Asian patients would largely benefit from the development of NOACs. Furthermore, the usage of NOACs has constantly increased while aspirin prescription has gradually decreased among Asians in recent years [10]. Hence, the rise of NOACs has revolutionized the field of NVAF management for Asian patients [11].

Until now, several NOACs (dabigatran, rivaroxaban, apixaban and edoxaban) have been approved in Asian countries, and it is still unclear that which NOAC is more effective and safer. Although direct or indirect comparisons between NOACs have been published, these trails mainly focus on western countries and results from Asia are considerably limited. Considering the ethnic and regional differences, it is important to assess the most favourable oral anticoagulation for Asian patients. Randomized controlled trial (RCT) represents the gold standard for evaluating the clinical effectiveness of an intervention. However, results from real-world studies could perfectly complement RCTs due to the RCT’s ability to reflect a genuine clinical practice and sample larger populations [12]. Comparison based on high-quality real-world studies is an alternative option in the absence of large RCTs.

Hence, the objective of this study is to conduct a network meta-analysis (NMA) comparing the clinical efficacy and safety of several NOACs in clinical practice among Asian patients with NVAF.

## Methods

### Search strategy

The literature search was implemented by two investigators in Embase, Medline, and the Cochrane Library from the databases’ inceptions through March 1, 2019. Search terms included atrial fibrillation, apixaban, dabigatran, rivaroxaban, edoxaban, real world, observational studies, and registry studies. The reference lists was also screened for included studies and relevant reviews to increase the sample size of literature reviewed. Only articles published in English were selected in this study for quality control. The detailed search strategies were presented in Additional file 4: Table S1.

### Study selection

The population, interventions, comparisons, outcomes, and study design (PICOS) were used to define the eligibility criteria. The inclusion criteria were as follows: (1) Asian patients with NVAF; (2) treatments with NOACs (apixaban, dabigatran, rivaroxaban or edoxaban) for stroke prevention; (3) real-world studies including prospective or retrospective cohort studies; (4) adjusted hazard ratio (HR) using propensity score matching (PSM) or multivariate analysis. The following studies were excluded: (1) Asian patients with valvular AF or non-Asian patients; (2) treatment with aspirin (±clopidogrel); (3) non-full-text or studies not published in English; (4) case-control studies or cross-section studies.

Two investigators reviewed literature separately and evaluated the included articles by pre-specified selection criteria. Disagreements were resolved through discussions between the two investigators or consulting with a third investigator.

### Data extraction

Two investigators independently extracted the effective information regarding the study design, treatments, patient characteristics, the number of enrolled patients, follow-up duration, and outcomes including SSE, ischemic strokes, all-cause deaths, major bleedings, and intracranial haemorrhages.

### Quality assessment

Assessment of the risk of bias for included studies was performed using the Newcastle-Ottawa Quality Assessment Scale (NOS) which is recommended by the Cochrane Collaboration for observational studies [13, 14]. The NOS has eight items within three domains: selection (representativeness), comparability (due to design or analysis), and outcomes (assessment and follow-up). A study scores one star for the satisfaction of each criterion, with the exception of the comparability domain (design or analysis), where a maximum of two stars can be awarded. In this study, publications that achieved eight or more stars on the NOS were considered as high quality, and moderate quality was defined as the achievement of six to seven starts, and less than six starts were considered low quality.

### Statistical analysis

Network meta-analysis was conducted using the mvmeta software package in STATA14 software. NMA synthesizes data from a network of trials and provides an integration of direct evidence with indirect evidence producing a relative ranking of all treatments [15], which is able to provide a solution to the present challenge of few head to head comparisons between different NOACs in Asians.

Adjusted HR for primary and secondary outcomes were estimated in this NMA. Primary outcomes included stroke or systemic embolism and major bleeding; four secondary outcomes were ischemic strokes, all-cause deaths, major bleedings, and intracranial haemorrhages. The hierarchy of the treatments was performed using the surface under the cumulative ranking (SUCRA) curve, where a larger SUCRA value represented a better rank of the treatment [16].

Statistical heterogeneity will be assessed with *P* values and I^2^ statistics (percentage of total variation across studies due to heterogeneity). An I^2^ value over 50% indicates substantial heterogeneity while an I^2^ value under 50% indicates low or moderate heterogeneity. Either a random-effect or fixed-effect model was adopted based on the result of heterogeneity analysis. Network inconsistency was evaluated by an inconsistency plot to examine the differences between direct and indirect evidence. Potential publication bias was assessed through visual inspection of funnel plots.

### Sensitivity analysis

Although potential confounders in these studies included in this NMA were adjusted using PSM or multivariate analysis, it is plausible that there are more confounding variables in real-word studies, especially in low-quality studies which included relatively smaller populations with inadequate comparability. Hence, a sensitivity analysis was conducted by excluding studies with low quality to improve the reliability of results from this NMA.

## Results

### Systematic literature review

**Fig. 1:**
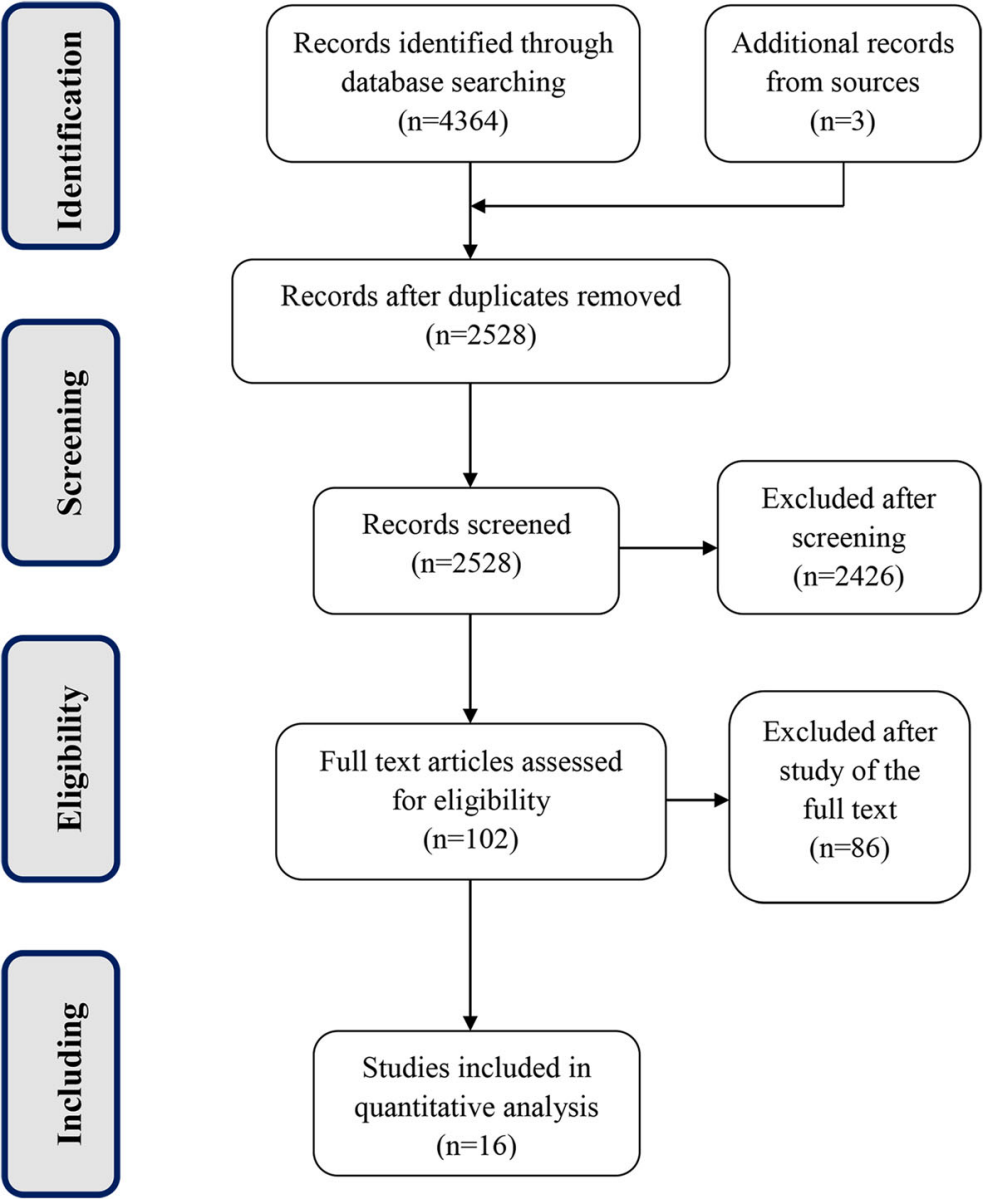
Preferred Reporting Items for Systematic Reviews and Meta-Analyses flow diagram

We identified a total of 4,367 records through database searching and other sources, and 2,528 records remained after the removal of duplicates. Following a screening of the title and abstracts, 2,426 records were eliminated and 102 full-text publications were further assessed for inclusion (Fig. 1). Sixteen studies [17–32] which evaluated the efficacy or safety of apixaban, rivaroxaban, and dabigatran with warfarin were included.

### Study characteristics

A sample size of 312,827 Asian patients receiving NOACs or warfarin therapies were included in ten real-world studies (Table 1). The baseline characteristics of the selected studies were summarized in Table 1. Seven studies were conducted in Taiwan; four studies in Japan and three in Korea; two studies based in both Hong Kong and Malaysia. Of these, eleven studies used propensity score methods to balance covariates across groups. Three studies included patients with a high risk of thromboembolism, or a CHA2DS 2-VASc score ≥ 4, while the other studies scored a range from 2.32 to 3.98.

**Table 1:** Baseline Characteristics of the Included Studies

Study	Country	Treatment	Sample Size (*n*)	Age (years)	Males (%)	CHADS2 –VASc score	History of stroke/TIA (%)	Hypertension (%)	Renal dysfunction (%)	Heart failure (%)	Diabetes (%)
Ho 2012 [17]	Hong Kong	Dabigatran	122	70.00	55.7	2.48	43.4	69.7	9	25.4	28.7
		Warfarin	122	70.01	52.5	2.32	32.0	63.1	13	31.1	34.4
Yap 2016 [18]	Malaysia	Dabigatran	500	65.3	62	2.69	NR	68.4	NR	6.2	31.4
		Warfarin	500	66.8	61.2	3.40	NR	75.6	NR	25.6	43.2
Chan 2016 [19]	Taiwan	Dabigatran	9940	75	58	4.13	33	87	23%	16	41
		Warfarin	9913	76	58	4.16	33	87	23%	16	41
Chan 2016 [20]	Taiwan	Rivaroxaban	3916	76	46	4.12	29	87	22%	16	41
		Dabigatran	5921	75	58	4.08	32	86	22%	16	41
		Warfarin	5251	71	56	3.32	20	75	21%	16	36
Cha 2017 [21]	Korea	Rivaroxaban	5681	70.5	52.7	3.60	NR	75.7	NR	44.3	23.8
		Dabigatran	3741	69.3	58.0	3.57	NR	76.8	NR	45.0	26.5
		Apixaban	2189	70.3	54.4	3.51	NR	76.9	NR	43.0	23.6
		Warfarin	23222	68.82	56.9	3.57	NR	76.9	NR	51.3	26.1
Naganuma 2017 [22]	Japan	Dabigatran	181	69	72	3.0	29	61	NR	18	29
		Warfarin	181	69	72	3.1	31	64	NR	15	29
Lai 2017 [23]	Taiwan	Rivaroxaban	4600	75.4	54.8	3.3	19.5	49.7	NR	NR	20.2
		Dabigatran	4600	75.4	54.6	3.3	19.1	49.4	NR	NR	20.4
Kohsaka 2017 [24]	Japan	Rivaroxaban	6726	75.8	62.0	3.3	22.3	53.8	4.4	35.3	28.9
		Dabigatran	5090	73.1	65.9	3.0	19.9	50.9	2.5	28.9	27.6
		Apixaban	5977	77.4	59.4	3.5	22.4	54.0	7.3	38.7	27.9
		Warfarin	14037	77.6	60.1	3.6	22.3	55.9	13.3	43.1	29.6
Hsu 2017 [25]	Taiwan	Rivaroxaban	300	75.2	44.7	NR	35.7	92.3	43.3	41.3	100
		Dabigatran	305	75.1	56.4	NR	33.8	91.8	38.4	40.3	100
		Warfarin	1899	70.0	50.9	NR	35.5	87.2	38.5	46.5	100
Deitelzweig 2017 [26]	Japan	Rivaroxaban	11082	77.2	52.5	4.4	11.5	NR	NR	NR	NR
		Dabigatran	2474	76.8	55.1	4.3	10.9	NR	NR	NR	NR
		Apixaban	8250	78.0	51.5	4.6	11.8	NR	NR	NR	NR
		Warfarin	14051	78.2	55.2	4.7	15.8	NR	NR	NR	NR
Lee 2018 [27]	Taiwan	Rivaroxaban	732	74.68	61.34	3.9	19.95	85.93	36.07	20.22	46.86
		Dabigatran	535	73.57	65.98	3.82	21.5	86.54	29.91	21.50	46.36
		Apixaban	171	75.36	55.56	3.98	20.47	87.72	41.52	18.71	43.86
		Warfarin	946	72.41	63.70	3.66	18.24	83.52	83.52	22.29	44.89
Huang 2018 [28]	Taiwan	Rivaroxaban	9637	75.20	54.56	4.02	26.25	73.74	11.72	35.35	31.00
		Warfarin	9637	74.98	54.70	4.11	26.97	74.02	11.97	36.77	31.79
Chan 2018 [29]	Taiwan	Rivaroxaban	27777	75	55	3.83	20	86	24%	13	39
		Dabigatran	20079	75	60	3.74	24	84	20%	11	38
		Apixaban	5843	76	55	3.89	20	87	29%	13	41
		Warfarin	19375	71	58	3.26	15	78	24%	14	36
Jeong 2019 [30]	Korea	Rivaroxaban	804	71.4	63.3	3.3	29.2	53.5	NR	5.7	24.1
		Warfarin	804	70.4	60.4	3.4	29.2	54.7	NR	5.1	22.3
Koretsune 2019 [31]	Japan	Dabigatran	4606	74	66	3.3	13	NR	NR	35	29
		Warfarin	4606	73	66	3.3	13	NR	NR	35	29
Cho 2019 [32]	Korea	Rivaroxaban	21000	73.8	50.9	3.6	21.1	87.8	NR	20.5	45.5
		Dabigatran	12593	72.9	53.6	3.5	24.3	87.0	NR	18.0	45.5
		Apixaban	12502	74.3	47.7	3.7	24	86.7	NR	20.6	45.3
		Warfarin	10409	70.8	54.0	3.5	27.3	85.9	NR	22.8	48.4

Age and follow-up duration reported in mean or median. MI = myocardial infarction; TIA = transient ischemic attack; NR = no results

### Quality assessment and sensitivity analysis

Quality evaluation was conducted using NOS (Additional file 5: Table S2) and most of the included studies were assessed as high-quality evidence (*N* = 12). Nevertheless, two studies were considered as low quality and a sensitivity analysis was consequently performed by discarding the two studies.The results of bias risk assessment and inconsistency test were presented in Additional file 1: Figure S1, Additional file 2: Figure S2 and Additional file 3: Figure S3.

Sensitivity analysis was conducted to test the stability of this NMA by excluding two low-quality studies. The results of sensitivity analysis were roughly consistent with the finding of the overall results.

### SSE and ischemic stroke

**Fig. 2:**
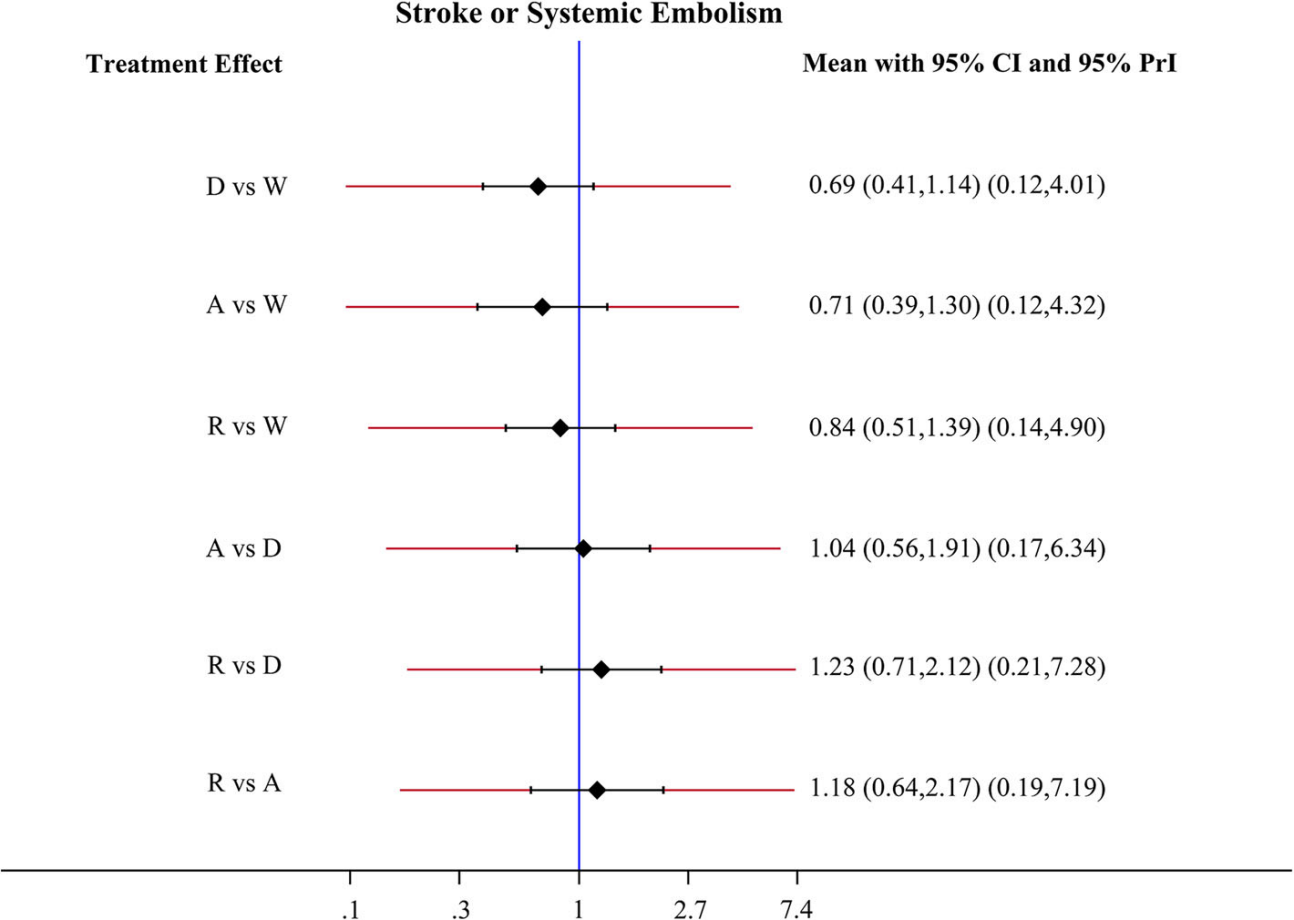
Forest plots for the primary efficacy outcome stroke and systemic embolism. Abbreviations: W = warfarin; A = apixaban; D = dabigatran; R = rivaroxaban

**Fig. 3:**
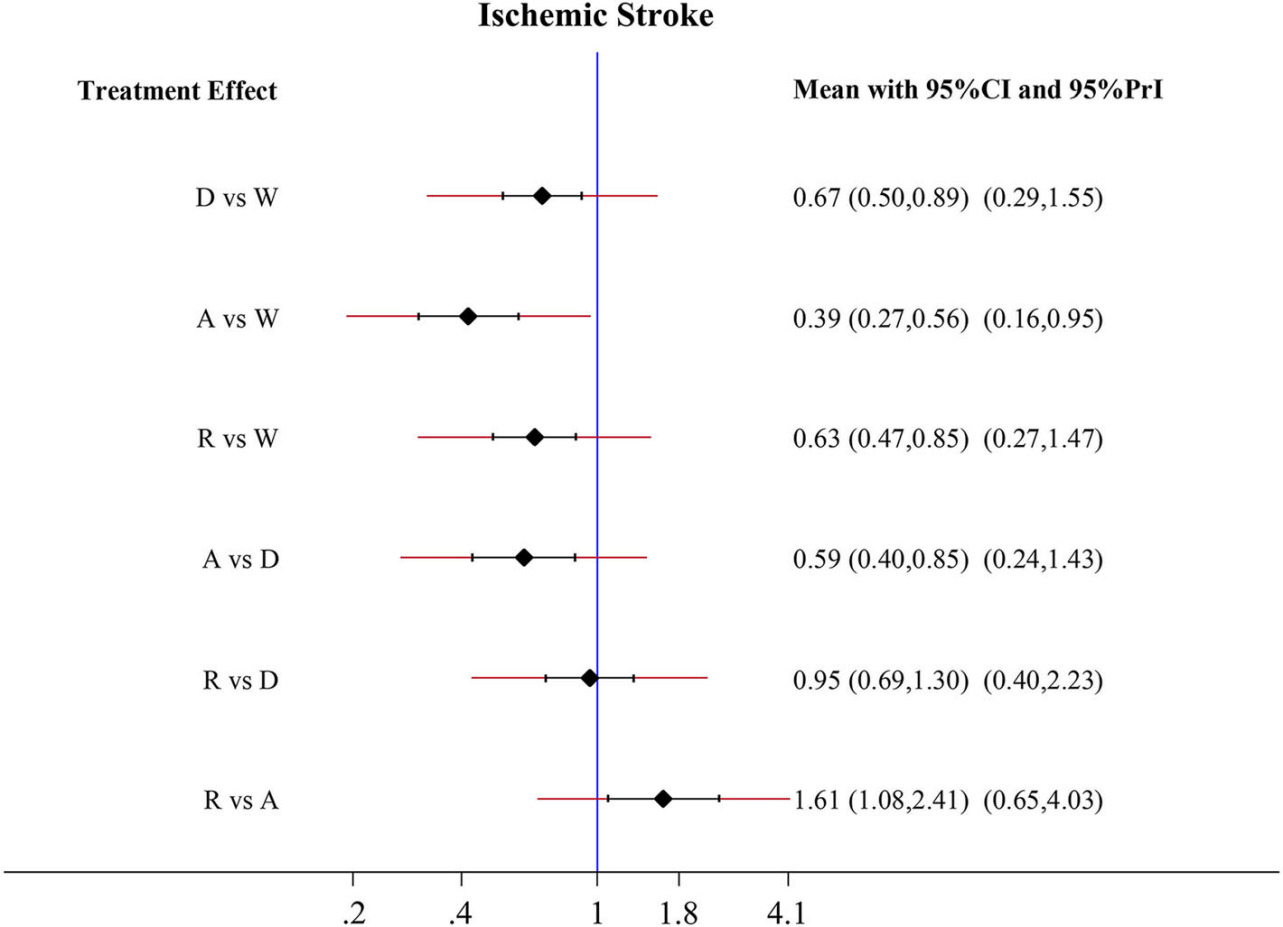
Forest plots for the secondary efficacy outcome ischemic stroke. Abbreviations: W = warfarin; A = apixaban; D = dabigatran; R = rivaroxaban

Apixaban, dabigatran, and rivaroxaban had a tendency to reduce the risk of SSE compared to warfarin, although no statistical significance was observed (Fig. 2). Apixaban, dabigatran, and rivaroxaban significantly reduced the risk of ischemic stroke in comparison to warfarin (apixaban: HR 0.39, 95% CI 0.27–0.56; dabigatran: HR 0.67, 95% CI 0.50–0.89; rivaroxaban: HR 0.63, 95% CI 0.47–0.85) (Fig. 3). In comparison to warfarin, Apixaban reduced the risk of ischemic stroke by 61%. Moreover, apixaban (HR 0.59; 95% CI 0.40–0.85) was superior to dabigatran in lowering stroke risk, meanwhile rivaroxaban (HR 1.61; 95% CI 1.08–2.41) was associated with a higher risk of ischemic stroke than apixaban. However, there was no significant difference between apixaban and dabigatran.

### Major bleeding

**Fig. 4:**
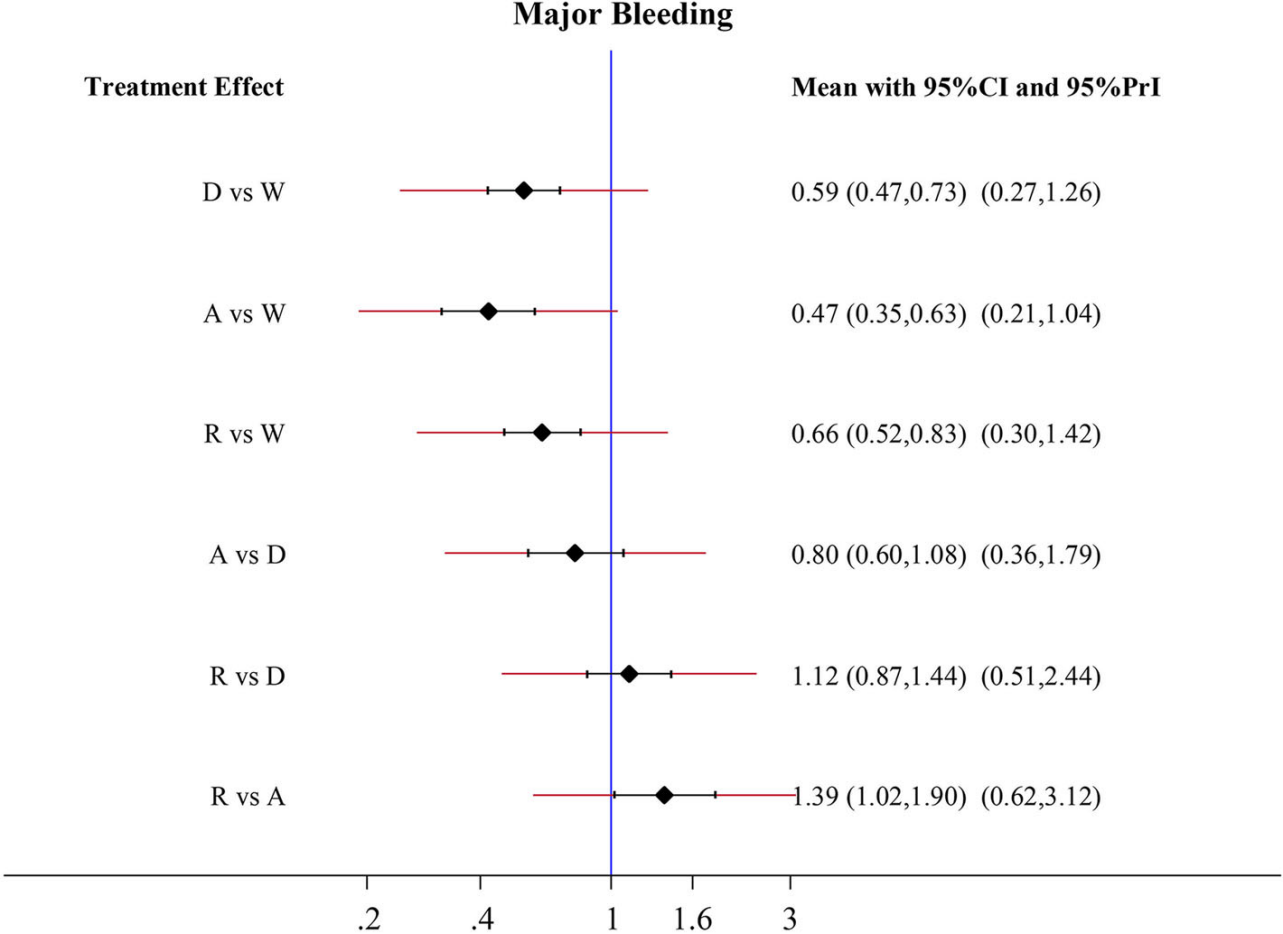
Forest plots for the primary safety outcome major bleeding. Abbreviations: W = warfarin; A = apixaban; D = dabigatran; R = rivaroxaban

According to the analysis, apixaban, dabigatran and rivaroxaban were excellent in lowering the major bleeding complications when compared to warfarin (apixaban: HR 0.47, 95% CI 0.35–0.63; dabigatran: HR 0.59, 95% CI 0.47–0.73; rivaroxaban: HR 0.66, 95% CI 0.52–0.83) (Fig. 4). Furthermore, rivaroxaban had a higher risk and caused major bleeding (HR 1.39, 95% CI 1.02 1.90) than apixaban. In addition, there was no significant difference between apixaban and dabigatran.

### Intracranial bleeding

**Fig. 5:**
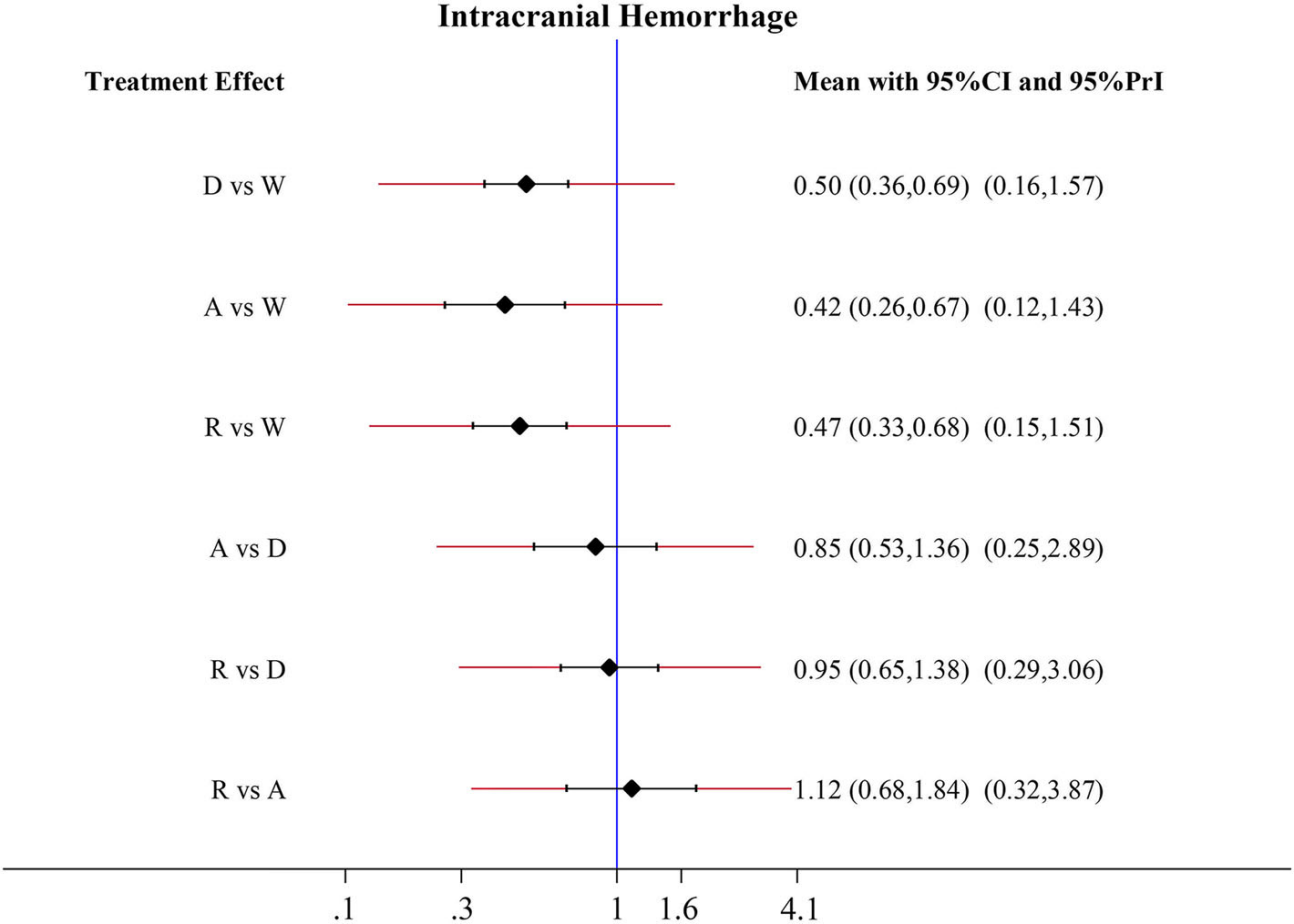
Forest plots for the secondary safety outcome Intracranial bleeding. Abbreviations: W = warfarin; A = apixaban; D = dabigatran; R = rivaroxaban

Apixaban, dabigatran, and rivaroxaban showed lower risks of intracranial bleeding when compared to warfarin ranged from 50 to 58% (apixaban: HR 0.42, 95% CI 0.26–0.67; dabigatran: HR 0.50, 95% CI 0.36–0.69; rivaroxaban: HR 0.47, 95% CI 0.33–0.68) (Fig. 5). There was no significant difference between apixaban, dabigatran, and rivaroxaban.

### All-cause death

**Fig. 6:**
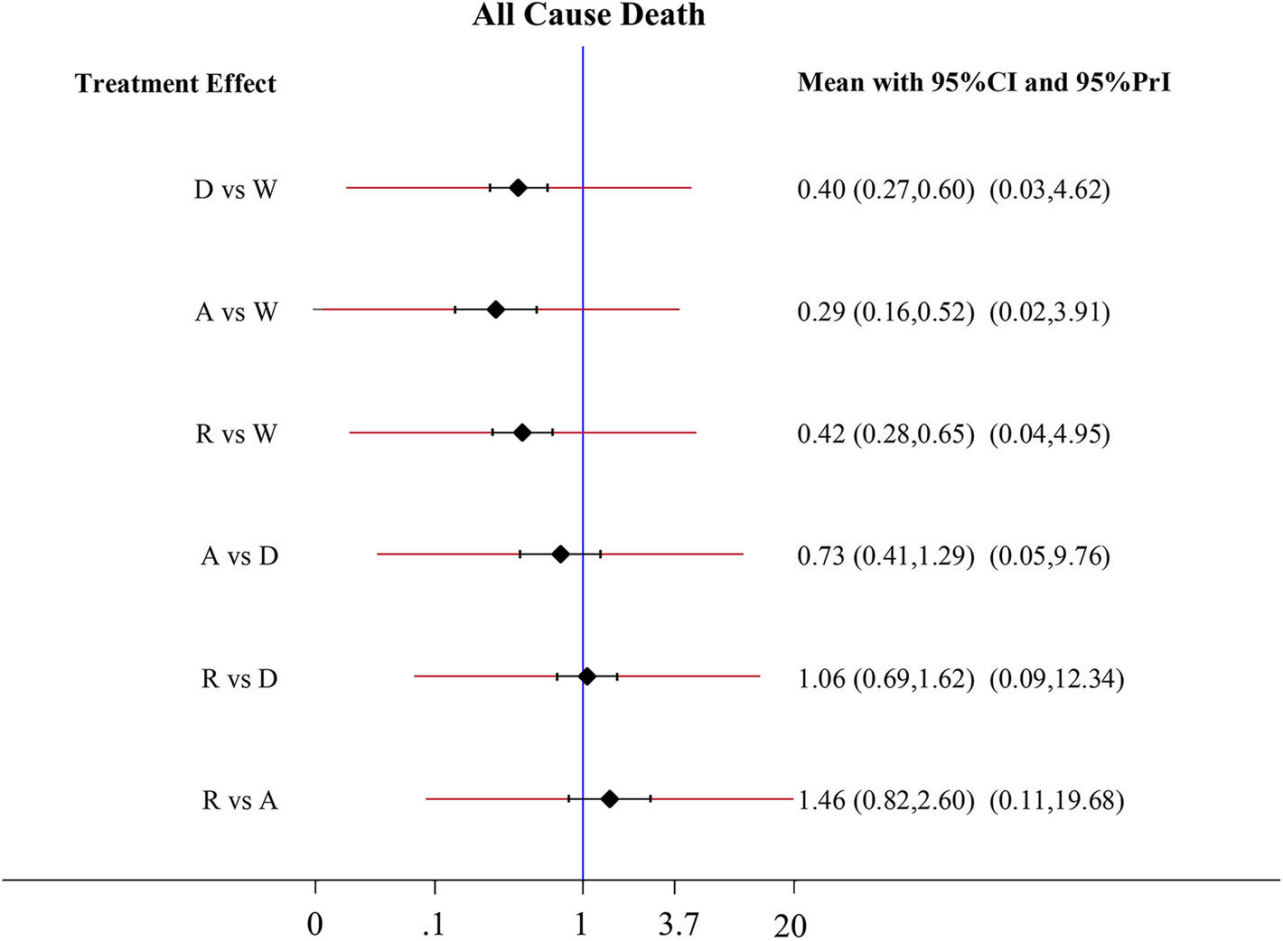
Forest plots for the secondary efficacy outcome All-cause death. Abbreviations: W = warfarin; A = apixaban; D = dabigatran; R = rivaroxaban

Apixaban (HR 0.29; 95% CI 0.16–0.52), dabigatran (HR 0.40; 95%CI 0.27–0.60) and rivaroxaban (HR 0.42; 95% CI 0.28–0.65) offered a significant advantage over warfarin at lessening all-cause death rate with a reduction ranging from 58 to 71% (Fig. 6). No significant difference between the three NOACs was observed.

### Clustered ranking of treatments

**Fig. 7:**
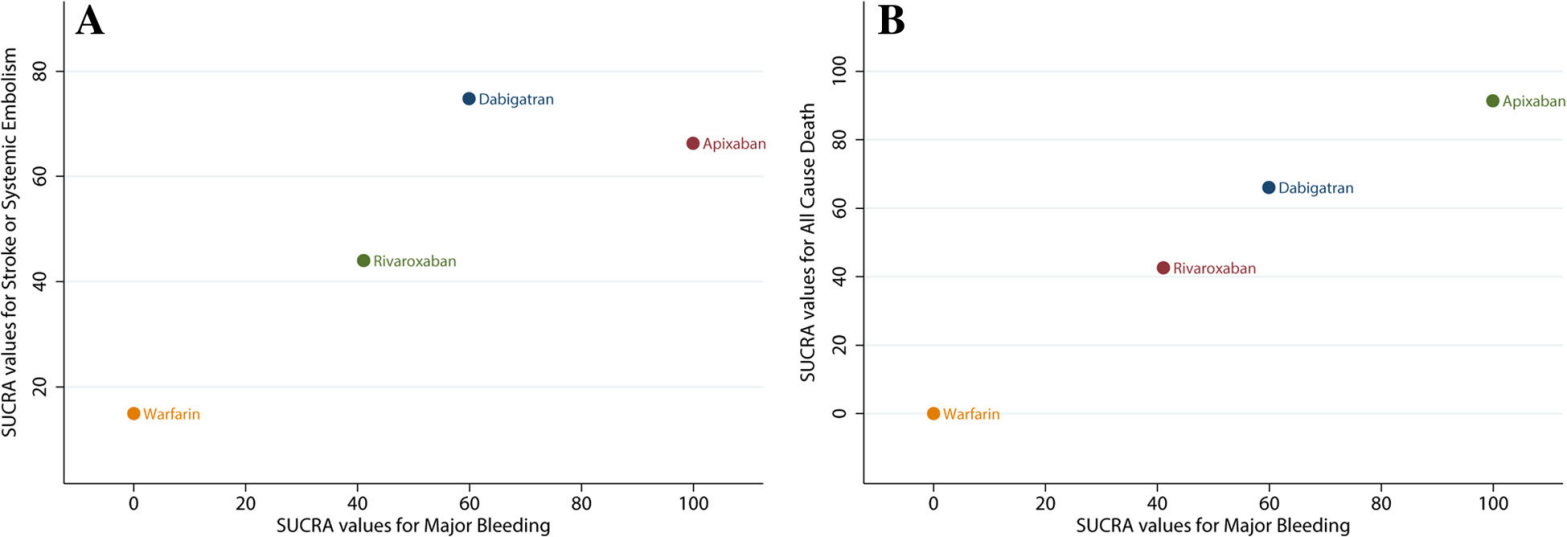
Clustered ranking plots. Surface under the cumulative ranking (SUCRA) curves values were used to represent the probabilities of each treatment being ranked best in each endpoint. Treatments lying in the upper right corner have more efficacy and are safer than the other treatments

Clustered ranking plots combined efficacy and safety endpoints (SSE, all-cause death, and major bleeding) based on SUCRA values and evaluated the optimal oral anticoagulants for Asian patients. Clustered ranking for SSE and major bleeding indicated that apixaban and dabigatran performed better compared to rivaroxaban (Fig. 7a). In the clustered ranking plots of all-cause death and major bleeding, apixaban demonstrated a good balance in both safety and efficacy endpoints (Fig. 7b).

## Discussion

Despite the numerous meta-analysis or NMA conducted in the field, the direct or indirect comparisons between NOACs focusing on Asian patients remain elusive. Through the NMA, we overcame the barrier of lacking head to head evidence in Asian patients and conducted an indirect comparison of several NOACs among patients with non-valvular atrial fibrillation. The present analysis suggested that apixaban, dabigatran, and rivaroxaban were superior to warfarin in reducing the risks of stroke and haemorrhage. Comparing with dabigatran, apixaban was associated with a lower risk of ischemic stroke, but no statistically significant difference was observed in terms of clinical safety. Moreover, we found that rivaroxaban was associated with a higher risk of major bleeding and ischemic stroke when compared with apixaban. However, there was no statistically significant difference in both safety and efficacy outcomes between rivaroxaban and dabigatran.

A recent study [9] suggested that NOACs as a whole were superior to warfarin in lowering stroke and bleeding risks. The present analysis comparing three NOACs (apixaban, rivaroxaban and dabigatran) with warfarin, further suggested that apixaban, rivaroxaban, and dabigatran were safer and more effective in stroke prevention among Asians. In comparison with warfarin, apixaban can lower the risk of ischemic stroke by 61% (Fig. 3) and all-cause death rate by 71% (Fig. 6). Apixaban, rivaroxaban, and dabigatran also cause less bleeding compared to warfarin, which may be related to the high risk of warfarin-induced intracranial bleeding and the low quality of INR control in Asia [33, 34].

Previous studies [4, 35–37], which mainly included the studies from western countries, suggest no significance in efficacy between apixaban and dabigatran, but less major bleeding risk arises from the apixaban usage. However, the present analysis suggests that apixaban provides an additional 41% reduction on ischemic stroke risk (Fig. 3) without showing an improved safe outcome, which may be partly due to the commonly low-dose usage of dabigatran in clinical practice among Asian patients [18, 22]. In agreement with previous studies [4, 36–38], apixaban had less major bleeding complications compared with rivaroxaban (Fig. 4). Furthermore, the present analysis demonstrates that rivaroxaban can increase the chance of ischemic stroke when compared to apixaban among Asians patients. However, due to an insufficient study sample size selected for this analysis, further research is required in order to draw a more conclusive result.

Evidence from RCTs is usually considered more reliable than real-world studies such as in the following studies. Nevertheless, performing an RCT requires strict eligibility criteria within a relatively small patient population, which limits the generality of results to be used as a clinical guide [39]. Real-world studies can estimate a much broader population not limited to age and other diseases, while being closer to a clinical practice [40]. Thus, real-word studies are considered a potential alternative to complement the evidence from RCTs [41]. Furthermore, propensity score matching (PSM) is widely performed in recent real-world studies to balance the distribution of biases and confounders between groups in order to achieve the purpose of simulating random assignment [12]. The majority of studies in this NMA performed PSM or multivariate analysis to maximally eliminate the influence of confounding factors. Although the results of this analysis are somewhat different from previous investigations, the findings still reflect the practical clinical benefits of oral anticoagulants among Asians patients. Moreover, the results from clinical practice could provide a new perspective on the use of NOACs for Asians.

In this analysis, we found that low-dose NOACs, especially dabigatran, were more widely used in clinical practice among Asian patients [18, 22], although a recent study [42] suggests that standard dose NOACs are more effective in stroke prevention without increasing risks of bleeding for Asians. However, considering the increasing complexity and variety of clinical practices, such as serious comorbidities and advanced age, it is understandable that low-dose oral anticoagulants are more likely to be prescribed by clinicians in order to avoid severe bleeding complications [5, 6, 43]. A recent observational study from Korea suggested that both dose dabigatran displayed similar efficacy outcomes; moreover, dabigatran 110 mg performed better than dabigatran 150 mg with regards to lowering bleeding risk [44]. At present analysis, we also found that although most of the Asian patients in this NMA received low-dose dabigatran (110 mg), dabigatran can effectively minimize the danger of ischemic stroke and major bleeding compared with warfarin.

Several limitations are present in this analysis. Firstly, the initial aim of this analysis was to compare the differences between four NOACs with each other, including apixaban, dabigatran, rivaroxaban, and edoxaban, but the studies that compared edoxaban with other anticoagulants for NVAF in the East Asia-Pacific region is limited. Thus, this analysis mainly studied the differences among apixaban, dabigatran, and rivaroxaban. Secondly, this NMA merely evaluated the mixed dose oral anticoagulants and the difference between regular and reduced dose was not tested mainly due to the lack of studies which analyzed the different doses of NOACs separately in Asia [25, 12]. Therefore, further studies are expected to be conducted to comprehensively understand the impact of dosage of oral anticoagulants on Asians. Thirdly, the studies included in this analysis were mainly from East Asian and Southeast Asian countries and regions, namely Taiwan, Japan, and Korea. Studies from the other parts of Asia, especially larger populated countries such as India and Pakistan, were missing due to the language barrier since only English literature were selected for this study. Nevertheless, considering the great quality control of publications in English journals due to the peer-review process, the bias is likely to be minimal.

## Conclusion

In conclusion, the NMA for Asians with NVAF suggested apixaban, dabigatran, and rivaroxaban were more effective than warfarin on reducing the risks of stroke and haemorrhage; apixaban appeared to demonstrate lower risks of stroke and haemorrhage comparing to rivaroxaban. However, considering of the limitation of observational study, these results need to be further comfirmed in rigorous head-to-head RCTs.

## Additional files

Additional file 1: **Figure S1.** Funnel plots. (A) Stroke or Systemic Embolism; (B) Ischemic stroke; (C) Major bleeding; (D) Intracranial bleeding; (E) All-cause death. (TIF 348 kb)

Additional file 2: **Figure S2.** Inconsistency plots. (A) Stroke or Systemic Embolism; (B) Ischemic stroke; (C) Major bleeding; (D) Intracranial bleeding; (E) All-cause death. (TIF 268 kb)

Additional file 3: **Figure S3.** Contribution plots. (A) Stroke or Systemic Embolism; (B) Ischemic stroke; (C) Major bleeding; (D) Intracranial bleeding; (E) All-cause death. Abbreviations: W = warfarin; A = apixaban; D = dabigatran; R = rivaroxaban. (TIF 464 kb)

Additional file 4: **Table S1.** Search Strategy. (DOCX 17 kb)

Additional file 5: **Table S2.** Evidence Quality Assessment in the Included Studies. (DOCX 21 kb)

## Data Availability

Not applicable.

## Abbreviations

AF: Atrial fibrillation
HR: Hazard ratio
INR: International normalized ratio
NMA: Network meta-analysis
NOACs: Novel oral anticoagulants
NOS: Newcastle-Ottawa quality assessment scale
NVAF: Non-valvular atrial fibrillation
PSM: Propensity score matching
RCT: Randomized controlled trial
SSE: Stroke or systemic embolism
SUCRA: Surface under the cumulative ranking curve
