# Supplementary figures and images for: Indirect comparison of novel Oral anticoagulants among Asians with non-Valvular atrial fibrillation in the real world setting: a network meta-analysis
Source: BMC Cardiovasc Disord. 2019 Jul 31;19:182. doi: 10.1186/s12872-019-1165-5 (PMC6670242; doi:10.1186/s12872-019-1165-5)

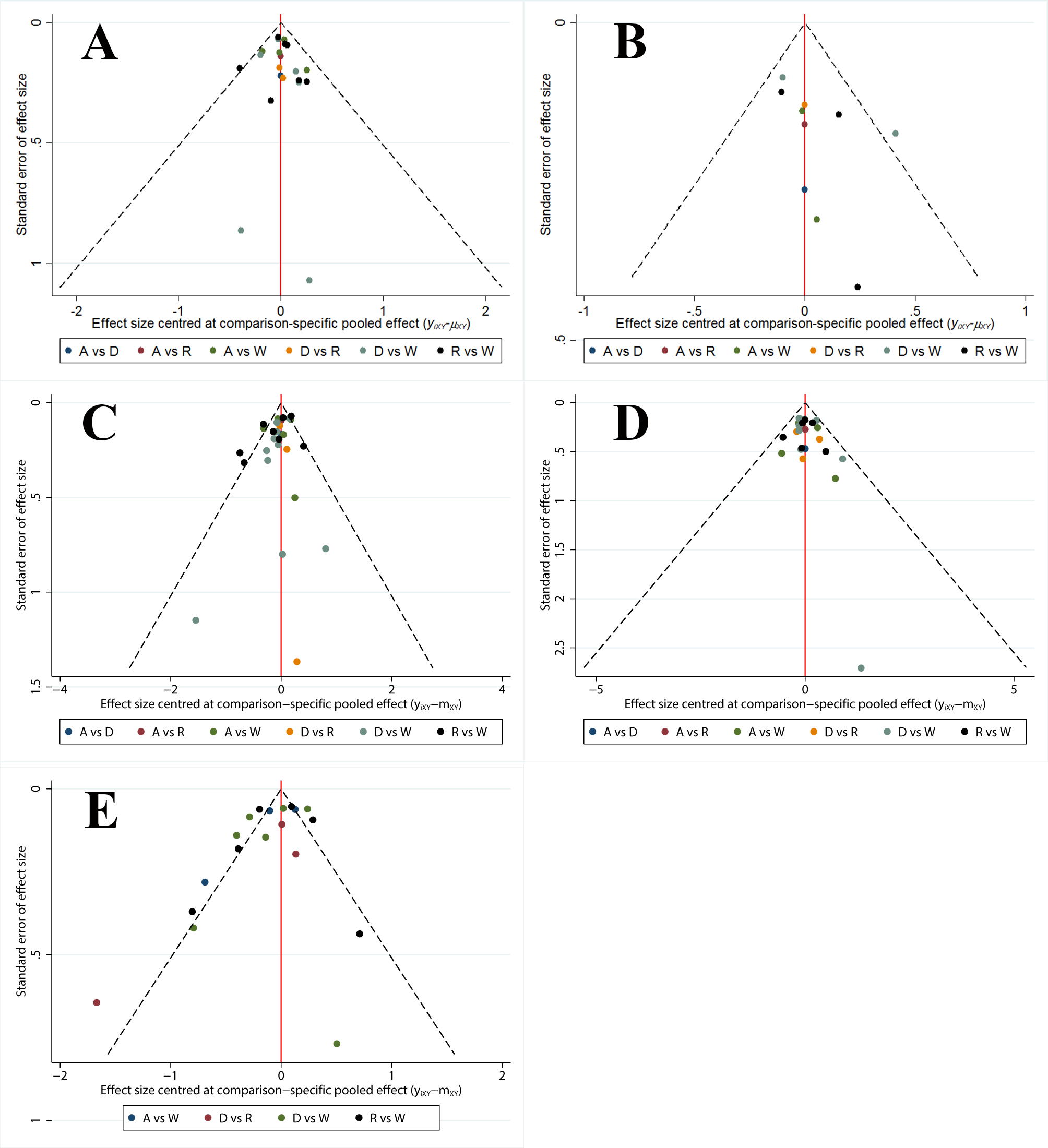

Supplement: Supplementary file 1 — Figure S1. Funnel plots. (A) Stroke or Systemic Embolism; (B) Ischemic stroke; (C) Major bleeding; (D) Intracranial bleeding; (E) All-cause death. (TIF 348 kb) [file 12872_2019_1165_MOESM1_ESM.tif]

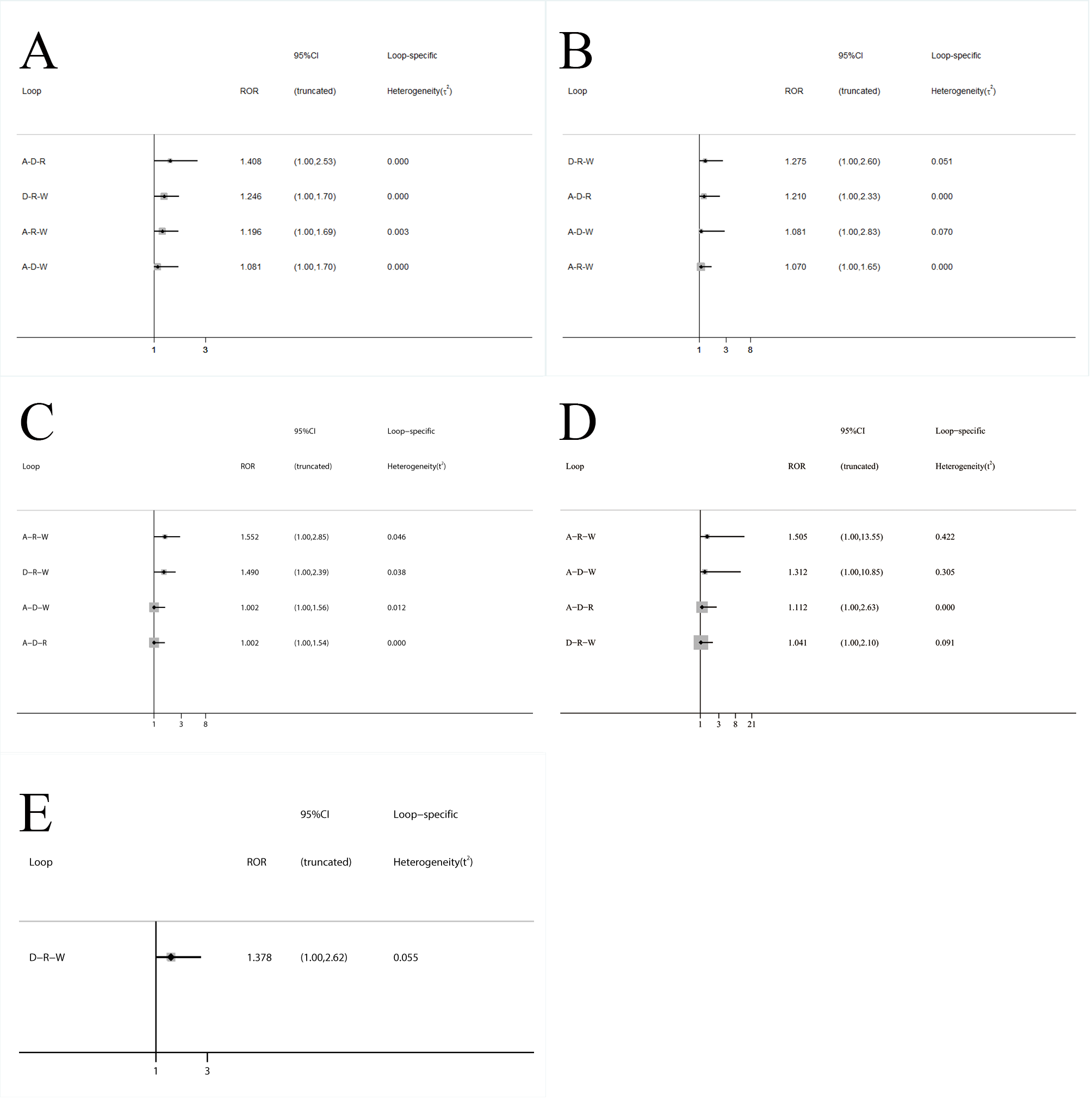

Supplement: Supplementary file 2 — Figure S2. Inconsistency plots. (A) Stroke or Systemic Embolism; (B) Ischemic stroke; (C) Major bleeding; (D) Intracranial bleeding; (E) All-cause death. (TIF 268 kb) [file 12872_2019_1165_MOESM2_ESM.tif]

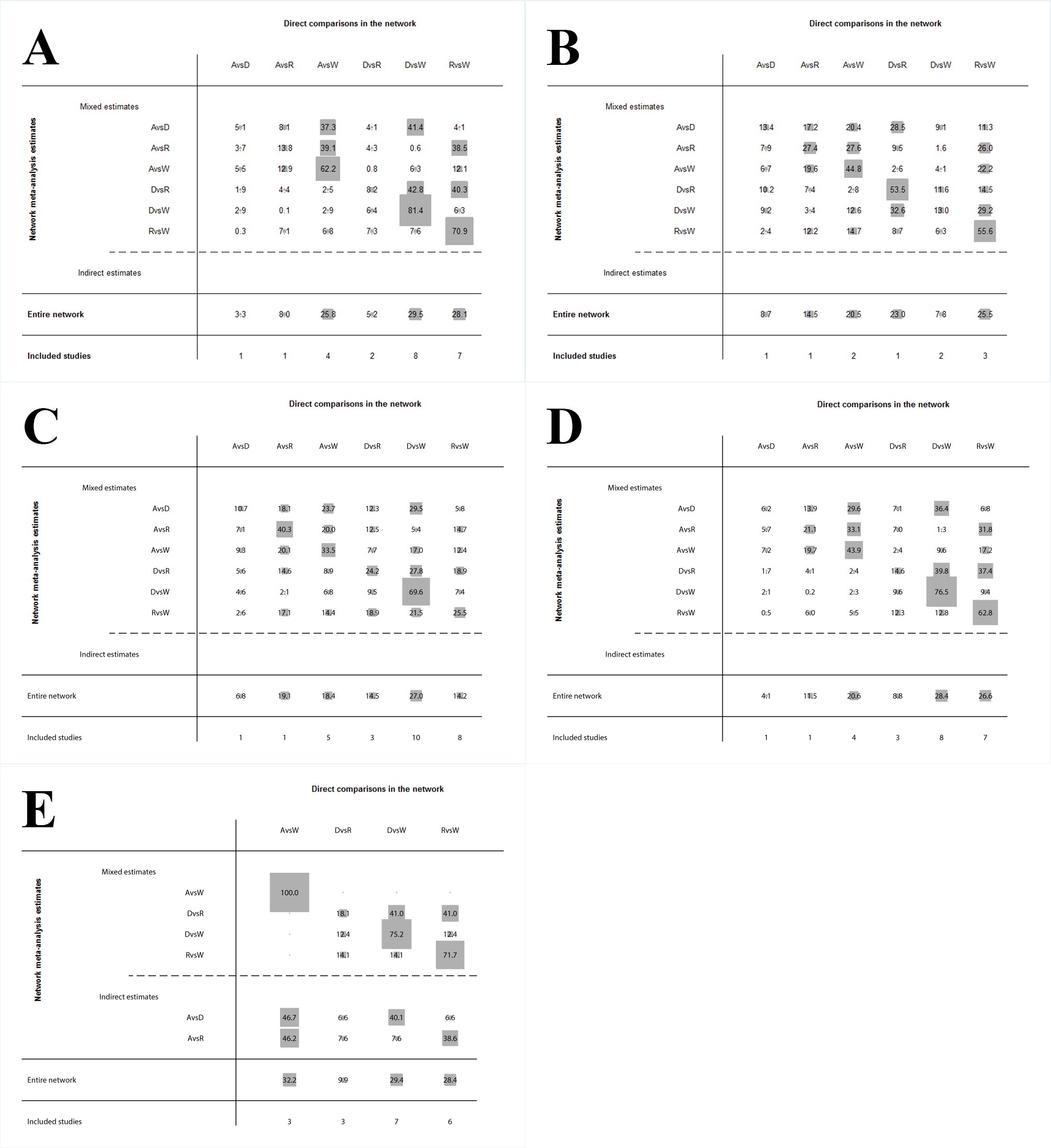

Supplement: Supplementary file 3 — Figure S3. Contribution plots. (A) Stroke or Systemic Embolism; (B) Ischemic stroke; (C) Major bleeding; (D) Intracranial bleeding; (E) All-cause death. Abbreviations: W = warfarin; A = apixaban; D = dabigatran; R = rivaroxaban. (TIF 464 kb) [file 12872_2019_1165_MOESM3_ESM.tif]
