# Supplementary material for: Indirect comparison of novel Oral anticoagulants among Asians with non-Valvular atrial fibrillation in the real world setting: a network meta-analysis
Source: BMC Cardiovasc Disord. 2019 Jul 31;19:182. doi: 10.1186/s12872-019-1165-5 (PMC6670242; doi:10.1186/s12872-019-1165-5)
Supplement: Supplementary file 4 — Table S1. Search Strategy. (DOCX 17 kb) [file 12872_2019_1165_MOESM4_ESM.docx]

**Table S1. Search Strategy**

**Embase and MEDLINE Search Strategy**

#1. ('atrial fibrillation' OR 'non-valvular atrial fibrillation')/exp

#2. ((atrial OR atrium OR auricular) NEAR/5 (fibrillation OR arrhythmia* OR flutter*)):ab,ti

#3. #1 OR #2

#4. (direct* NEAR/5 thrombin NEAR/5 inhib*):ab,ti

#5. (factor* NEAR/5 Xa NEAR/5 inhib*):ab,ti

#6. (rivaroxaban OR apixaban OR dabigatran OR edoxaban):ab,ti

#7. ((new or novel) NEAR/5 ‘oral anticoagulant*’):ab,ti

#8. (‘non-vitamin K antagonist*’ OR ‘NOAC*’ OR ‘Direct oral anticoagulant*’):ab,ti

#9. #4 OR #5 OR #6 OR #7 OR #8

#10. (‘real world*’ OR observational OR registr*):ab,ti

#11. ‘cohort study’/exp

#12. #10 OR #11

#13. #3 AND #9 AND #12

**COCHRANE Search Strategy**

#1. MeSH descriptor Atrial Fibrillation, this term only

#2. MeSH descriptor Atrial Flutter, this term only

#3. (atrial or atrium or auricular) near5 (fibrillation* or arrhythmia* or flutter*):ti,ab,kw

#4. (AF):ti,ab,kw

#5. (#1 OR #2 OR #3 OR #4)

#6. (direct* adj5 thrombin adj5 inhib*):ti,ab,kw

#7. (factor* adj5 Xa adj5 inhib*):ti,ab,kw

#8. (rivaroxaban OR apixaban OR dabigatran OR edoxaban):ti,ab,kw

#9. ((new or novel) adj5 (oral anticoagulant*)):ti,ab,kw

#10. (non-vitamin K antagonist* OR NOAC* OR Direct oral anticoagulant*):ti,ab,kw

#11. #6 OR #7 OR #8 OR #9 OR#10

#12. #5 AND #11
