# Supplementary material for: Indirect comparison of novel Oral anticoagulants among Asians with non-Valvular atrial fibrillation in the real world setting: a network meta-analysis
Source: BMC Cardiovasc Disord. 2019 Jul 31;19:182. doi: 10.1186/s12872-019-1165-5 (PMC6670242; doi:10.1186/s12872-019-1165-5)
Supplement: Supplementary file 5 — Table S2. Evidence Quality Assessment in the Included Studies. (DOCX 21 kb) [file 12872_2019_1165_MOESM5_ESM.docx]

| **Study** | **Selection** | | | | **Comparability of cohort** | **Outcome** | | | **Evidence quality** |
| --- | --- | --- | --- | --- | --- | --- | --- | --- | --- |
|  | **Exposed cohort representative** | **Non-exposed cohort selection** | **Exposure ascertainment** | **Outcome not present at start** |  | **Assessment** | **Follow-up length** | **Follow-up adequacy** |  |
| Ho 2012^[17]^ | * | * | * | - | * | * | - | - | Low |
| Yap 2016^[18]^ | * | * | * | - | * | * | - | - | Low |
| Chan 2016^[19]^ | * | * | * | * | ** | * | * | * | High |
| Chan 2016 ^[20]^ | * | * | * | * | ** | * | * | * | High |
| Cha 2017^[21]^ | * | * | * | * | ** | * | - | * | High |
| Naganuma 2017^[22]^ | * | * | * | - | ** | - | * | * | Moderate |
| Lai 2017^[23]^ | * | * | * | * | ** | * | - | * | High |
| Kohsaka 2017^[24]^ | * | * | * | - | ** | * | * | * | High |
| Hsu 2017^[25]^ | - | * | * | - | ** | * | * | * | Moderate |
| Deitelzweig 2017^[26]^ | * | * | * | * | ** | * | - | * | High |
| Lee 2018^[27]^ | * | * | * | * | ** | * | * | * | High |
| Huang 2018^[28]^ | * | * | * | * | ** | * | * | * | High |
| Chan 2018^[29]^ | * | * | * | * | ** | * | * | * | High |
| Jeong 2019^[30]^ | * | * | - | * | ** | * | * | * | High |
| Koretsune 2019^[31]^ | * | * | * | * | ** | * | - | * | High |
| Cho 2019^[32]^ | * | * | * | * | ** | * | * | * | High |

**Table S2. Evidence Quality Assessment in the Included Studies**

Studies that achieved eight or more stars on the NOS were considered high quality and moderate quality was defined to achieve six to seven starts and less than six starts were considered as low quality.
